# Fatigue in intensive care unit survivors with COVID-19: An observational cohort study

**DOI:** 10.1016/j.bbih.2025.100943

**Published:** 2025-01-03

**Authors:** Kristina Struksnes Fjone, Milada Hagen, Jon Henrik Laake, Luis Romundstad, Eirik Alnes Buanes, Kristin Hofsø

**Affiliations:** a Department of Research and Development, Division of Emergencies and Critical Care, Oslo University Hospital, Oslo, Norway; b Department of Public Health, Oslo Metropolitan University, Oslo, Norway; c Section for Physiotherapy, Department of Clinical Services, Division of Cancer Medicine, Oslo University Hospital, Oslo, Norway; d Department of Anaesthesia and Intensive Care Medicine, Division of Emergencies and Critical Care, Oslo University Hospital, Oslo, Norway; e Lovisenberg Diaconal University College, Oslo, Norway; f Norwegian Intensive Care and Pandemic Registry, Haukeland University Hospital, Bergen, Norway; g Department of Postoperative and Intensive Care Nursing, Division of Emergencies and Critical Care, Oslo University Hospital, Oslo, Norway

## Abstract

• Fatigue was highly prevalent one year after ICU admission in ICU survivors with COVID-19.
• Younger age, pain/discomfort, dyspnoea, and mental health symptoms were associated with reports of fatigue.
• COVID-19 ICU survivors should be assessed with comprehensive symptom screening during follow-up care.

## 1 Background

Severe cases of Coronavirus disease 2019 (COVID-19) often present, as acute respiratory failure (ARF) which may progress to acute respiratory distress syndrome (ARDS) (Tan et al., 2021). An early report of patient characteristics from China, demonstrated that 22% of patients admitted to the hospital were transferred to the ICU because of ARDS (Wang et al., 2020). However, a recent publication estimated the pooled prevalence of COVID-19-induced ARDS in the global population to be 32.2%, demonstrating the severe consequences of COVID-19 (Azagew et al., 2023). ARDS is associated with high mortality rates in the intensive care unit (ICU), and with long-lasting symptoms that persist beyond the initial phase of critical illness (Bellani et al., 2016; Herridge et al., 2016). Patients with ARF and ARDS are at high risk of developing a complex set of symptoms known as post-intensive care syndrome (PICS) (Herridge et al., 2016; Rawal et al., 2017). PICS encompasses the physical, psychological, and cognitive symptoms that ICU patients experience following treatment (Needham et al., 2012; Elliott et al., 2014). During the COVID-19 pandemic, the term “post-COVID-19 condition” was introduced to describe health challenges characteristic of patients with COVID-19. The term is defined by the presence of symptoms lasting a minimum of two months, with no attributable aetiology from other identifiable medical conditions (World Health Organization, 2021). Post-COVID-19 condition represents a broader definition, including a more comprehensive range of symptoms that not only apply to patients with critical illness. During the pandemic, fatigue emerged as one of the most frequently reported symptoms in patients with post-COVID-19 condition (Lopez-Leon et al., 2021). Fatigue is not, however, explicitly addressed within the PICS framework. Fatigue is defined in various ways and is described in conjunction with many medical conditions (Billones et al., 2021). It is often described as extreme and persistent tiredness, exhaustion, or weakness that is not relieved by rest, and can include multiple dimensions, such as physical, cognitive, and emotional fatigue (Dittner et al., 2004). The COVID-19 pandemic has raised awareness about this issue as numerous studies on COVID-19 describe fatigue as the most frequent long-lasting symptom regardless of illness severity (Lopez-Leon et al., 2021; Gesser et al., 2023). Fatigue can impair patient's ability to resume work or education and interferes with the rehabilitation process (Souron et al., 2021; Hosey et al., 2021). Although some studies lack conclusive evidence on the association between illness severity and persistent fatigue, other studies have identified significant associations between ICU admission and the prevalence and severity of fatigue in COVID-19 patients (Gesser et al., 2023; Darcis et al., 2021; Huang et al., 2022; Sigfrid et al., 2021; Townsend et al., 2020). It is, therefore, crucial to further investigate this potentially debilitating symptom in ICU populations with COVID-19. Overall, there is limited understanding of the prevalence and severity of fatigue in both general ICU populations and the COVID-19 population, including underlying mechanisms, risk factors, symptom duration, and symptom trajectories (Spadaro et al., 2020).

Hence, the overall aim of this study is to describe the prevalence of fatigue and identify factors associated with fatigue in COVID-19 survivors during the first year following ICU admission.

## 2 Methods

### 2.1 Study design and setting

We conducted this prospective observational cohort study as a collaboration between the Norwegian Intensive Care and Pandemic Registry and Oslo University Hospital. This study is part of a larger observational study (NCT04601090) aimed at describing survival rates and long-term outcomes in COVID-19 ICU patients in Norway. Detailed information about the Norwegian Intensive Care and Pandemic Registry has been published in separate studies presenting results on post-traumatic stress symptoms and cognitive impairment (a) (Fjone et al., 2024a, Fjone et al., 2024b). The present study adheres to the Strengthening the Reporting of Observational Studies in Epidemiology (STROBE) guidelines for comprehensive and transparent reporting of observational studies (von Elm et al., 2007).

### 2.2 Study sample

The study sample was selected from the Norwegian Intensive Care and Pandemic Registry. All survivors above 18 years of age at admission to any Norwegian ICU between March 1, 2020 and June 30, 2021, with COVID-19 (confirmed by polymerase chain reaction (PCR) test), were eligible for inclusion. The study sample represents patients admitted during the first three waves of the pandemic in Norway. ICUs reporting to the registry need to meet all the following criteria: 1) have a defined area for ICU treatment, 2) be equipped and monitored for ICU, 3) have ICU-proficient nurses and doctors, and 4) treat ICU patients on a daily basis. From these units, survivors must meet one or more inclusion criteria for registration: 1) length of stay (LOS) ≥ 24 h, 2) requirement for ventilator support, 4) transfer to another ICU within 24 h, or 5) need for a continuous infusion of vasoactive substances [ (Buanes et al.), p. 159]. The Norwegian Intensive Care and Pandemic Registry collects data from all major ICUs in Norway and has an estimated data completeness rate of 92% [ (Buanes et al.), p. 166]. Patients who could not read, write, or understand Norwegian were excluded from the study as only Norwegian questionnaires were administrated from the registry.

### 2.3 Data collection

The study utilised clinical and demographic data, along with patient-reported outcome measures (PROM) from the registry. Additionally, supplemental questionnaires were collected by the study group using telephone interviews. The registry collected data on fatigue, self-perceived lung function, pain/discomfort, and health-related quality of life (HRQoL) at both 3 and 12 months following ICU admission. Additionally, the study group collected data on anxiety and depression at 12 months post-ICU admission. The registry also collected data on post-traumatic stress symptoms (PTSS) at 12 months follow-up. The data were collected through secure electronic platforms (Helsenorge or Digipost) or mail. The study group contacted patients by telephone or by mail if telephone number was unknown. Patients received a reminder from the registry or the study group if there was no response after one month.

### 2.4 Main outcome

The main outcome of this study was fatigue, assessed using the Chalder Fatigue Scale (CFQ) (Chalder et al., 1993). The CFQ consists of 11 items, with a score ranging from 0 (less than usual) to 3 (much more than usual) for each item, and a total score from 0 to 33 (Cella and Chalder, 2010). The scale includes seven items measuring physical fatigue and four items measuring mental fatigue (Cella and Chalder, 2010). The CFQ can be used as a continuous scale, with higher scores indicating more severe fatigue, or as a bimodal scale (Cella and Chalder, 2010). In bimodal scoring, the two lower categories are coded as 0 and the two highest categories as 1, resulting in a total score from 0 to 11. A cut-off of ≥4 indicates a case of fatigue (Cella and Chalder, 2010). There is no consensus regarding a cut-off for the continuous score; therefore, we used bimodal scoring in this study to investigate the dichotomous outcome, e.g. the prevalence of fatigue at 3 and 12 months following ICU admission. To investigate the associations between fatigue and possible predictive factors, we used both the bimodal and continuous scores in separate analyses. The CFQ has been translated into Norwegian, and Norwegian normative data are available (Loge et al., 1998). Although, the instrument is not validated for ARF or ICU patients, it has been used in studies involving hospitalised COVID-19 patients (Bellan et al., 2021; O'Brien et al., 2022). In this study, all items demonstrated high internal consistency (Cronbach's Alpha >0.90) at both 3- and 12-month follow-ups).

### 2.5 Clinical and demographic variables

Clinical data from the ICU included predefined risk factors for developing severe illness of COVID-19 (e.g., diabetes, cardiovascular disease, smoking), a severity of illness measure (Simplified Acute Physiology Score (SAPS) II) (Le Gall et al., 1993), ICU treatment details (e.g., mechanical ventilation (MV)), ICU length of stay (LOS), and frailty score (Clinical Frailty Scale) (Rockwood et al., 2005). Peripheral oxygen saturation and respiration rate were recorded upon admission to the emergency department. Demographic data included age, sex, and self-reported education and cohabitation.

### 2.6 Self-reported symptoms/variables

#### 2.6.1 Pain/discomfort

The EQ-5D-5L is a generic questionnaire that contains two parts (Devlin et al., 2020; Feng et al., 2021). The first part includes five statements covering five domains: mobility, self-care, usual activities, pain/discomfort, and anxiety/depression (Devlin et al., 2020). Each domain is rated from 1 (no problems) to 5 (extreme problems). In this study, the pain/discomfort item was used to investigate the association between pain/discomfort and fatigue in the regression models. The second part of the instrument comprises the EQ VAS, which provides an assessment of the patient's overall health, rated from 0 (worst health imaginable) to 100 (best health imaginable). However, this assessment was not used in the present study (Devlin et al., 2020). The EQ-5D-5L has demonstrated excellent psychometric properties in a wide range of populations (Feng et al., 2021). It has been translated into Norwegian, and Norwegian normative data are available (Garratt et al., 2022).

#### 2.6.2 Anxiety and depression

Anxiety and depression were measured using the Hospital Anxiety and Depression Scale (HADS), which consists of 14 items: seven for anxiety and seven for depression (Snaith, 2003). Each item is scored on a scale from 0 (not at all) to 3 (very much), resulting in a total score ranging from 0 to 21. The subscales for anxiety and depression are scored separately (Snaith, 2003). A cut-off value of ≥8 was used in this study, to indicate symptoms of anxiety or depression (Snaith, 2003). HADS has been widely used within the ICU population, has good psychometric properties (Needham, 2020), has been translated into Norwegian, and is validated for a Norwegian population (Mykletun et al., 2001).

#### 2.6.3 Symptoms of post-traumatic stress

Symptoms of post-traumatic stress were measured using the Impact of Event Scale-6 (IES-6), which consists of six items with scores ranging from 0 (not at all) to 4 (extremely), resulting in a total score ranging from 0 to 24 (Hosey et al., 2019). The cut-off score is calculated as the mean of all responses, with a score of ≥1.75 indicating symptoms of post-traumatic stress (PTS) (Hosey et al., 2019). The IES-6 is validated for ARDS patients and has good psychometric properties (Hosey et al., 2019). The IES-6 is an abbreviation of the Impact of Event Scale-Revised, which has been translated into Norwegian and validated for a Norwegian population (Thoresen et al., 2010; Eid et al., 2009).

#### 2.6.4 Dyspnoea

Self-perceived severity of breathlessness was measured using the modified Medical Research Council Dyspnea Scale (mMRC). This questionnaire uses a unidimensional scale to relate activities of daily living to dyspnoea through five different statements (Cotes, 1987). Participants were asked to respond to one of the statements, ranging from 0 (not troubled with breathlessness except on strenuous exercise) to 4 (too breathless to leave the house, or breathless when dressing or undressing). Although the mMRC is not validated for ICU patients, it has been used in multiple studies with hospitalised COVID-19 patients and is recommended for assessing dyspnoea in the post-COVID-19 condition (Gorst et al., 2023). Although it has been translated into Norwegian it has not been validated for a Norwegian population.

### 2.7 Statistical analyses

Descriptive data are presented as frequencies (counts) and proportions (percentages) for categorical variables, and as medians with ranges for continuous variables. To compare responders and non-responders, the Mann-Whitney *U* test was used for non-normally distributed continuous variables and the *t*-test for normally distributed continuous variables. Pairs of categorical data were compared using the Pearson Chi-Square test. Point estimates of the prevalence of fatigue are presented with 95% confidence intervals (CI), derived using binomial approximation, and are computed from the sample of responders at both time points. Possible associations between selected covariates and the outcome (fatigue) were analysed using a linear mixed-effects model with the continuous fatigue score as the dependent variable, for complete case analysis including responders at both the 3- and 12-month follow-ups to assess the effect of time. A logistic regression model was used for the 12-month follow-up analysis, with the dependent variable being fatigue score using the bimodal scoring. In univariate logistic regression analyses, each covariate was investigated for an association with the outcome variable. Variable selection for the univariate analyses was based on clinical and empirical considerations. Clinical factors included peripheral oxygen saturation and respiratory rate at hospital admission. Empirical factors encompassed demographics (age, sex), educational status, predefined risk factors, acute physiology (SAPS II score), pre-ICU frailty (Clinical Frailty Scale), length of ICU stay, duration of MV, and psychological well-being (PTSS, depression, anxiety, dyspnoea, and pain/discomfort). Multicollinearity was found between anxiety and depression, and between anxiety and PTSS. Anxiety was therefore excluded from the analyses. To explore possible confounding effects of PTSS on the other covariates, two different models were run: Model 1 included PTSS, and Model 2 excluded PTSS. Both models included all other remaining covariates. Variables associated with the dependent variable with a *p-*value ≤0.1 in the univariate analyses were included in the multivariate regression models. For the main outcome (fatigue), only responses without missing items were included in the analyses. The results from the linear mixed-effects model are presented as regression coefficients (B) with 95% CI. Odds ratios (OR) with 95% CI were estimated using logistic regression. Internal consistency in the CFQ was analysed using Cronbach's alpha. All tests were two-sided and *p*-values <0.05 were considered statistically significant. Data were analysed using IBM SPSS Statistics (version 29) and Stata (version 18).

### 2.8 Ethics

The study was approved by the Regional Committees for Medical and Health Research Ethics (reference number: 135310) and the institutional data protection officer. It followed the ethical principles for medical research described in the Declaration of Helsinki (World Medical Association Declaration of Helsinki, 2013). As the Norwegian Intensive Care and Pandemic Registry is a national registry, consent is not required. However, patients and/or their proxies received information regarding their registration, including the option to have their data deleted at any time. Participants who completed the telephone interviews provided oral consent before the interview and later confirmed their consent in writing.

## 3 Results

### 3.1 Patient characteristics

**Fig. 1:**
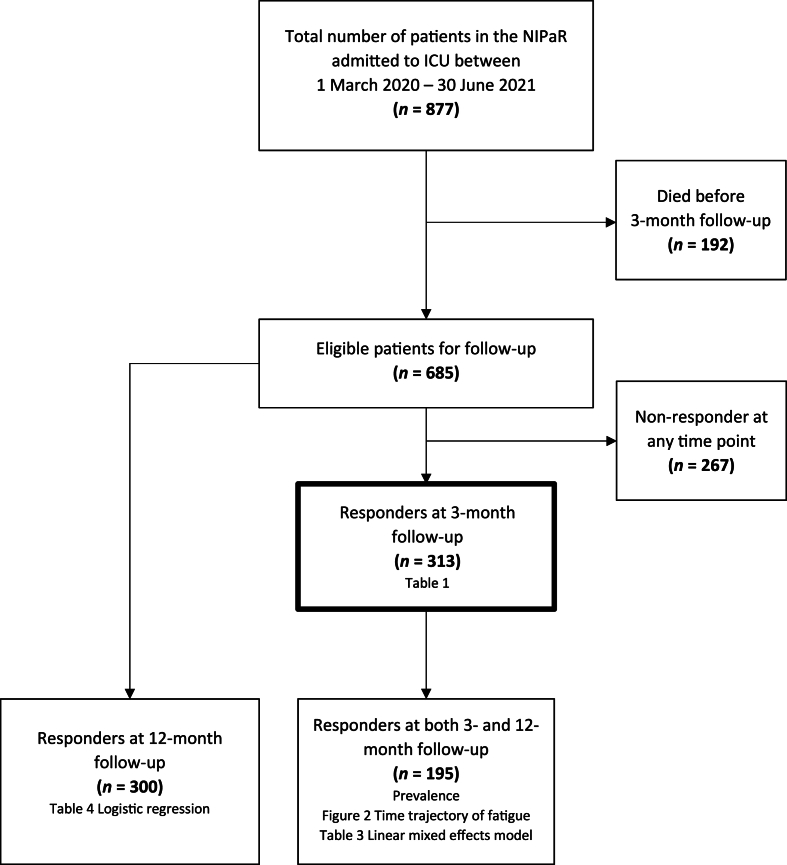
Flowchart.

Between March 1, 2020 and June 30, 2021, 877 patients with COVID-19 were admitted to Norwegian ICUs. Of these, 192 died before the three-month follow-up, leaving 685 eligible for the present study. A total of 313 patients responded to the CFQ at 3 months following ICU admission, and 300 patients responded to the 12-month follow-up. A total of 195 patients responded at both time points (Fig. 1 Flowchart).

Clinical and demographic characteristics at ICU admission for the study sample (*n* = 313) are shown in Table 1. The median age of study participants was 59 years (range 18–91), and the majority were male (70.6%). The median ICU LOS was 10 days (range 0.1–71.8), and 86.5% received MV (Table 1). There were no significant differences in demographic and clinical characteristics between the responders and non-responders at the 3- and 12-month assessments. The only difference between the responders and non-responders at any time point was their age at ICU admission, as the non-responders were younger than the responders (Table 2).

**Table 1:** Characteristics of the 3 month responders (*n* = 313).

Clinical characteristics at time of ICU admission	*n*	%	Median (range)
Age	313		59 (18─91)
Sex			
Female	92	29.4	
Male	221	70.6	
Clinical Frailty Scale	201		2 (1─7)
Peripheral oxygen-saturation (%)	277		90 (50─100)
Respiration rate (per minute)	302		26 (10─63)
SAPS II	313		30 (6─72)
ICU length of stay (days)	313		10 (0.1─71.8)
Received mechanical ventilation	271	86.5	
Time on mechanical ventilation (days)	271		7 (0.1─56.6)
Type of mechanical ventilation			
Time on invasive mechanical ventilation	160		11 (0.3─54)
Time on non-invasive ventilation	220		1.3 (0.1─11.7)
Received ECMO	3		
Any risk factors			
Yes	255	81.5	
No	58	18.5	
Risk factors^a^			
Cardiovascular disease	121	38.7	
Obesity	86	27.5	
Diabetes Mellitus I or II	47	15	
Asthma	45	14.4	
Chronic lung disease (asthma not included)	24	7.7	
Immune deficit	18	5.8	
Cancer	15	4.8	
Kidney disease	14	4.5	
Other^b^	14	4.5	

Demographics measured at 3 or 6 months	*n*	%
Education (*n* = 236)^c^		
Primary/secondary school	133	56.4
Higher education – College/university	103	43.6
Co-habitation (*n* = 238)^c^		
Living with someone	192	80.7
Living alone	46	14.7
EQ-5D-5L pain/discomfort item (*n* = 310)^d^		
No	58	18.7
Slight	134	43.2
Moderate	79	25.5
Severe	33	10.6
Extreme	6	1.9
mMRC (*n* = 294) d		
Not troubled	93	31.6
Short of breath	100	34
Walks slower than contemporaries	60	20.4
Stops for breath after walking 100 m	34	11.6
Too breathless to leave the house	7	2.4

a Some have more than one risk factor.

b Other include smoking, neurological disease, pregnancy, and liver disease.

c Measured at 6 months.

d Measured at 3 months.

**Table 2:** Characteristics of responders and non-responders.

	Responders at 3 or 12 months (*n* = 418)			Non-responders any time point (*n* = 267)			*p-value*
	*n*	%	median (range)	*n*	%	median (range)	
**Age**	418		60 (18─91)	267		57 (24─88)	**0.02**
**Sex**							0.77
Male	294	70.3		185	69.3		
Female	124	29.7		82	30.7		
**Risk factor**							0.53
Yes	339	81.1		209	78.3		
No	79	18.9		55	20.7		
**SAPS II**	418		30 (6─72)	267		29 (6─70)	0.72
**CFS**	264		2 (1─7)	143		3 (1─9)	0.06
**ICU LOS**	418		10.1 (0.1─76.2)	267		9 (0.7─4.8)	0.18
**MV duration**	418		7.9 (0.1─69.7)	266		7.5 (0.1─69.3)	0.56

	Responders at 3 months (*n* = 313)			Non-responders at 3 months (*n* = 372)			*p-value*
	*n*	%	median (range)	*n*	%	median (range)	
**Age**	313		59 (18─91)	372		58 (24─88)	0.30
**Sex**							0.72
Male	221	70.6		258	69.4		
Female	92	29.4		114	30.6		
**Risk factor**							0.50
Yes	255	81.5		293	79.4		
No	58	18.5		76	20.6		
**SAPS II**	313		30 (6─72)	372		30 (6─70)	0.80
**CFS**	201		2 (1─7)	206		3 (1─9)	0.08
**ICU LOS**	313		10 (0.1─71.8)	372		9.1 (0.17─6.2)	0.73
**MV duration**	271		7 (0.1─56.6)	313		7.8 (0.1─69.7)	0.54

	Responders at 12 months (*n* = 300)			Non-responders at 12 months (*n* = 385)			*p-value*
	*n*	%	median (range)	*n*	%	median (range)	
**Age**	300		60 (18─91)	385		58 (22─88)	0.21
**Sex**							0.25
Male	203	66.7		276	71.7		
Female	97	32.3		109	28.3		
**Risk factor**							0.84
Yes	240	80		308	80.6		
No	60	20		74	19.4		
**SAPS II**	300		30 (6─72)	385		30 (6─70)	0.20
**CFS**	188		2 (1─7)	219		3 (1─9)	0.38
**ICU LOS**	300		10.6 (0.1─76.2)	385		9.1 (0.3─74.8)	0.23
**MV duration**	255		8.2 (0.1─69.7)	329		7 (0.1─69.3)	0.22

*CFS -* Clinical Frailty Scale; *ICU LOS ─* intensive care unit length of stay; *MV ─* mechanical ventilation; SAPS - Simplified Acute Physiology Score II.

### 3.2 Prevalence and time trajectory of fatigue

**Fig. 2:**
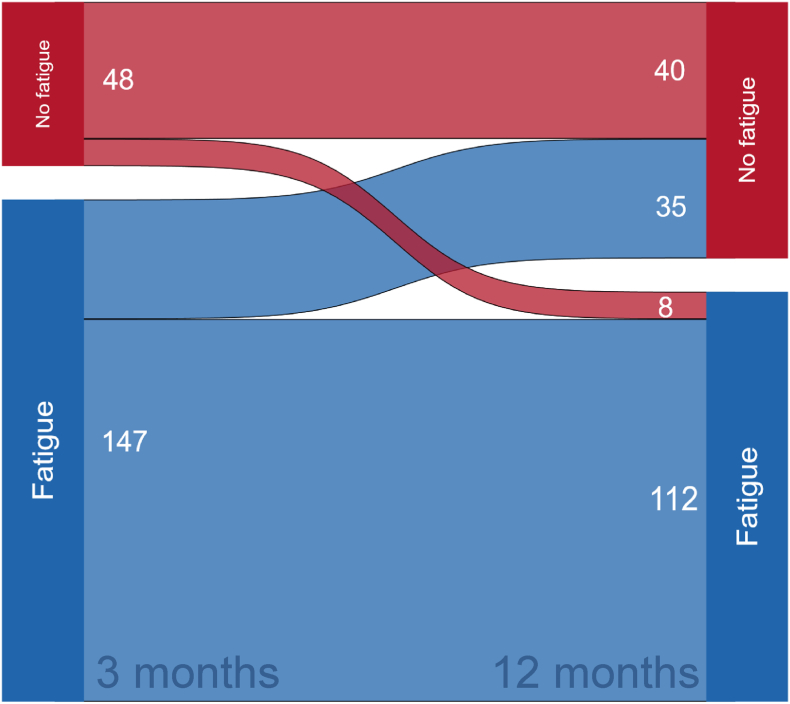
Time trajectory of fatigue.

For prevalence and longitudinal analyses, we analysed data on the 195 patients who responded at both 3- and 12-month follow-up following ICU admission. Of these patients, *n* = 147, 75.4% (95% CI [68.7−81.2]) scored ≥4 in the CFQ at 3 months, representing a case of fatigue, and at 12 months, *n* = 120, 61.5% (95% CI [54.3−68.4]) scored ≥4. The trajectories of fatigue from 3 to 12 months are presented in Fig. 2.

### 3.3 Factors associated with fatigue over time

Results from the linear mixed-effects model, including data for the 195 responders at both time points, are presented in Table 3. Fatigue decreased significantly over time, with scores at 12 months being 1.4 points lower compared to the assessment at 3 months (B = 1.40, 95% CI [0.12−2.65]). Younger age (B = −0.11, 95% CI [−0.16− −0.05]) was associated with significantly higher fatigue scores at both time points. Also, pain/discomfort (B = 2.40, 95% CI [1.61−3.20]), and dyspnoea (B = 1.37, 95% CI [0.65−2.10]) at 3 months were significantly associated with higher fatigue scores at both 3 and 12 months after ICU admission. This means that patients who reported pain/discomfort had proximately 2.4 points higher fatigue scores compared to those who did not report pain/discomfort. Similarly, patients reporting dyspnoea had nearly 1.4 points higher fatigue scores than those without dyspnoea.

**Table 3:** Linear mixed fixed effects model for fatigue between 3 and 12-months (*n* = 195).

		Fixed effects		
Variables		Estimate	95% CI	*p*-value
Time 3 months		1.40	0.12–2.65	**0.03**
Time 12 months	Reference			
Age		−0 0.11	−0.16–−0.05	**<0.001**
Sex	Male	−1.33	−2.78–0.12	0.07
	Female (reference)			
ICU LOS		0.03	−0.02–0.08	0.25
Clinical Frailty Scale		−0.48	−1.21–0.24	0.19
SAPS II		−0.01	−0.07–0.07	0.95
mMRC - dyspnoea		1.37	0.65–2.10	**<0.001**
EQ-5D-5L pain/discomfort		2.40	1.61–3.20	**<0.001**

*CI:* Confidence interval; *ICU LOS:* intensive care unit length of stay; *mMRC:* Modified Research Council Dyspnea Scale; *SAPS:* Simplified Acute Physiology Score II. Level of significance ˂0.05.

### 3.4 Factors associated with long-term fatigue

To further investigate the associations of long-term fatigue with possible predictive factors, we analysed all responders at 12 months (*n* = 300) using logistic regression (Table 4). In this sample, the prevalence of PTSS was 40.4% (*n* = 120/297), and the prevalence of depression was 16.7% (*n* = 36/215). In the multiple logistic regression Model 1, adjusted for age, sex, available risk factors, and selected clinical characteristics, PTSS (OR = 1.13, 95% CI [1.03─1.26]) was the only covariate that remained statistically significantly associated with long-term fatigue. This means that for each point increase in PTSS, patients were 13% more likely to report fatigue at 12 months. In our Model 2, which excluded PTSS as a covariate, younger age (OR = 0.96, 95% CI [0.93─0.99]), pain/discomfort (OR = 2.16, 95% CI [1.19─3.92]), and symptoms of depression (OR = 1.26, 95% CI [1.26─1.56]) were all statistically significantly associated with fatigue at 12 months. Our Model 1 results suggest that the effects of all covariates included in the model were likely confounded by PTSS, as the remaining covariates lost their statistical significance.

**Table 4:** Logistic regression analyses with and without PTSS as covariate. Predictive factors associated with scoring ≥4 in the CFQ at 12 months (*n* = 300).

				Model 1 with PTSS			Model 2 without PTSS		
	Univariate analyses			Multivariate analyses			Multivariate analyses		
	OR	95% CI	*p-*value	OR	95% CI	*p-*value	OR	95% CI	*p*-value
***During admission***									
Age	0.97	0.95─0.99	**<0.01**	0.98	0.94─1.01	0.18	0.96	0.93─0.99	**0.04**
Sex (ref. male)	2.22	1.31─3.76	**<0.01**	1.36	0.52─3.55	0.53	1.65	0.66─4.12	0.29
Risk factor (ref. no)	1.05	0.59─1.88	0.86						
SAPS II	0.99	0.97─1.01	0.47						
Clinical Frailty Scale	0.86	0.64─1.14	0.29						
ICU LOS (days)	1.01	0.99─1.03	0.11						
Duration of MV (hours)	1.01	0.99─1.04	0.20						
Peripheral oxygen-saturation	1.01	0.98─1.03	0.82						
Respiration rate (per minute)	1.01	0.98─1.04	0.41						
ECMO	∗	∗	∗						
***At* 12 months**									
EQ-5D-5L pain/discomfort item	3.50	2.37─5.17	**<0.001**	1.64	0.87─3.12	0.13	2.16	1.19─3.92	**0.01**
HADS depression (sum score)	1.65	1.40─1.94	**<0.001**	1.14	0.91─1.42	0.25	1.26	1.02─1.56	**0.03**
IES-6 (sum score)	1.29	1.21─1.37	**<0.001**	1.13	1.03─1.26	**0.02**			
Education (ref. lower)	0.38	0.22─0.65	**<0.001**	0.45	0.19─1.08	0.07	0.50	0.22─1.17	0.11
mMRC dyspnoea	2.96	2.11─4.16	**<0.001**	1.68	0.92─3.05	0.09	1.73	0.95─3.14	0.07

*CI:* Confidence interval; *ECMO:* Extracorporeal membrane oxygenation; *HADS*: Hospital Anxiety and Depression Scale; *ICU LOS:* intensive care unit length of stay; *mMRC:* Modified Research Council Dyspnea Scale; *MV:* mechanical ventilation; *OR:* Odds ratio; *SAPS:* Simplified Acute Physiology Score II. Level of significance ˂0.05. The Chalder Fatigue Scale score was used as a dichotomous dependent variable. ∗The model could not generate results due to the low number of patients receiving extracorporeal membrane oxygenation.

## 4 Discussion

In this cohort of ICU patients with COVID-19, the prevalence of fatigue declined significantly from 3 to 12 months following ICU admission, but it remained high, with 61.5% scoring above the CFQ cut-off at 12 months. In our study sample, 57.4% remained fatigued at both time points, while only 18% improved and scored below the cut-off at 12 months. These results confirm previous studies involving both COVID-19 ICU patients and general ICU patients, which report consistently high levels of fatigue and emphasise that fatigue is a key symptom for ICU survivors (Souron et al., 2021; Premraj et al., 2022; Bench et al., 2021; Heesakkers et al., 2022; Neufeld et al., 2020). Our results are consistent with the literature. For instance, a study from Sweden found that 64.4% of COVID-19 ICU survivors experienced fatigue one year after ICU admission (Hussain et al., 2022). Similarly, a study from the Netherlands reported a fatigue prevalence of 56.1% at 12 months following ICU treatment (Heesakkers et al., 2022).

Several studies have shown that fatigue is one of the most frequently reported symptoms in COVID-19 patients, regardless of illness severity, and some have suggested that illness severity does not influence the sensation of fatigue (Poole-Wright et al., 2023; Alkodaymi et al., 2022; Malik et al., 2022). Using the same cut-off as in the present study, a Norwegian cohort of non-hospitalised COVID-19 patients found that 46% experienced fatigue during the first six months after symptom onset, which is markedly lower than in the present study (Stavem et al., 2021). It should be noted that this study had a response rate below 50%, which could represent selection bias (Stavem et al., 2021). Interestingly, a pre-pandemic study with ARDS patients showed that 70% and 66% reported fatigue at 6- and 12-month follow-ups, respectively, following ICU treatment (Neufeld et al., 2020). To our knowledge, the latter study is the largest study of ICU patients assessing fatigue as the main outcome. It serves as a reminder that fatigue is not solely related to COVID-19 but may be more broadly associated with critical illness.

An issue of concern in the present study is that younger age was associated with long-term fatigue, as demonstrated in both the linear mixed model and logistic regression (Model 2) analyses. This finding aligns with another study involving COVID-19 ICU patients, where younger age was also identified as a risk factor for fatigue (Hussain et al., 2022). Given that fatigue can be a debilitating symptom, that prevents patients from returning to work or school and interfers with the rehabilitation process, addressing this issue is crucial (Souron et al., 2021). However, two recent systematic reviews and meta-analyses assessing fatigue in ICU patients and COVID-19 patients, respectively, have presented conflicting results, identifying both increased age and younger age as risk factors for reporting fatigue (Bench et al., 2021; Poole-Wright et al., 2023). Notably, the meta-analysis on COVID-19 patients included samples from mixed populations of non-hospitalised and hospitalised patients and has several limitations. Therefore, it is difficult to generalise its results to ICU patient populations (Poole-Wright et al., 2023). For several reasons, we hypothesise that younger people report more subjective complaints, such as fatigue. Their premorbid health is likely higher, meaning that severe illness leads to more significant relative functional loss. Also, younger individuals might have higher expectations for regaining previous health status than older individuals. Combining these two aspects could explain why younger persons tend to have a higher prevalence of subjective complaints. Thus, more research is needed to better understand the impact of age on the development of fatigue after critical illness.

Studies involving ICU patients often find non-modifiable variables such as age, sex, and prior mental health status to be associated with PICS (Lee et al., 2020). Understanding how different self-reported symptoms interact within ICU populations is limited, prompting us to present two models for the logistic regression analyses. In Model 1, PTSS seems to be a confounding factor, meaning that PTSS is also associated with all the other covariates in the regression model and they are consequently being confounded by PTSS. Therefore, PTSS is the only variable that remained statistically significantly associated with the outcome (fatigue) in the multiple logistic regression Model 1. In Model 2, symptoms of depression emerge as statistically significant, revealing an association between psychological distress and fatigue. This finding highlights the need to address mental health in patients presenting with fatigue during follow-up services after ICU admission. Depression is a well-known symptom associated with fatigue in various patient populations, not only in ICU patients (Neufeld et al., 2020; Løke et al., 2022). Several psychological phenomena (i.e., anxiety, depression, and PTSS) have overlapping symptoms and can be associated with both the outcome and other covariates. It is important to highlight that the direction of these associations is not yet fully understood. The co-occurrence of symptoms, their mutual influence, and their impact on various outcomes are well described in the Theory of Unpleasant Symptoms and were discussed in a previous publication from this study (Fjone et al., 2024b; Lenz et al., 2008). By presenting two models, we highlight that many ICU patients experience overlapping symptoms and underscore the importance of correct statistical modelling.

In the present study, we found statistically significant associations between fatigue and pain/discomfort when treating fatigue as both a continuous and categorical variable. Notably, patients who reported pain/discomfort had over twice the odds of having long-term fatigue. A recent study investigating causal relationships between fatigue, pain, and psychological distress found that pain and psychological distress had a strong direct impact on fatigue (Løke et al., 2022). The authors discuss the importance of always assessing and addressing multiple symptoms, including pain and psychological distress, when patients present with fatigue (Løke et al., 2022). We also found that self-reported dyspnoea was associated with fatigue over time. Other COVID-19 studies have found similar results, but most of these studies used samples from mixed populations of non-hospitalised and hospitalised participants, with few ICU patients included (Poole-Wright et al., 2023; Fernández-de-Las-Peñas et al., 2022). Therefore, comparisons between the present study, which includes only ICU patients, and previous studies with mixed populations should be made with caution. Additionally, dyspnoea is not always explained by lung function tests or chest imaging, as in the present study, indicating that the aetiology behind dyspnoea varies depending on whether it is based on self-report or objective tests (Lerum et al., 2023). The association between dyspnoea and fatigue has long been known, and muscle use is described as the most important physiological factor related to both dyspnoea and fatigue (Gift and Pugh, 1993). This is highly applicable to ICU patients who often present with reduced physical functioning and capacity long after discharge from the ICU (Souron et al., 2021). Our findings highlight the importance of addressing pain, dyspnoea, and mental health issues during clinical follow-up for ICU patients, as these symptoms can potentially be modified through multimodal interventions (Hosey et al., 2021). However, as discussed in the previous section, symptoms can interact with each other and be influenced by situational, psychological, and physiological factors. This interaction makes it difficult to determine the direction in which symptoms affect each other and complicates making causal conclusions (Lenz et al., 2008).

Most previously published COVID-19 studies have a follow-up period of 6 months after symptom onset or hospitalisation. However, there are clear indications that symptoms may persist even longer in both general ICU populations and COVID-19 ICU patients (Herridge et al., 2016; Huang et al., 2022). Fatigue can become persistent and chronic, potentially having a significant impact on quality of life (Bench et al., 2021). In a qualitative study assessing which symptoms were regarded as most important by patients and healthcare personnel during clinical follow-up after ICU treatment, fatigue emerged as the symptom that both groups considered essential to address (Nedergaard et al., 2018). We believe that the exploration of fatigue is still limited and that we lack an understanding of the magnitude of the problem, its causes, and possible mitigating factors. These factors need to be investigated in order to identify treatment options, both in the ICU and in the ICU follow-up care. Acknowledging that fatigue is a significant issue in post-ICU patients opens the door to managing it as a primary symptom that impacts cognitive and emotional recovery. By integrating fatigue assessments, providing energy management strategies, and tailoring rehabilitation plans, we can potentially improve cognitive function and reduce depressive symptoms. The present study provides further evidence and insights into long-term outcomes after ICU treatment, suggesting that fatigue should be specifically addressed within the PICS framework and included in a revised core outcome set for ICU patients.

### 4.1 Strengths and limitations

The response rate in the present study was 61% at any time point and 46% at both time points, which could be considered low. Even though we did not exclude many patients, excluding non-speaking Norwegians limits the study's generalisability. In Norway, which has a diverse demographic, this exclusion may disproportionately leave out specific populations, such as those with limited skills in Norwegian, who may also face barriers in accessing healthcare services. Furthermore, language skills might correlate with broader socioeconomic factors, including education, employment, and social integration. For instance, individuals from immigrant backgrounds may experience fatigue and psychological distress differently due to additional stressors related to language barriers, migration, or acculturation. During the early phases of the pandemic, immigrant populations and people in geographical areas with low socioeconomic status were over-represented. We wonder if most non-responses in the present study are due to insufficient understanding of Norwegian. The ability to compare responders and non-responders, along with multiple assessments in the same individuals, is a key methodological strength of this study. Additionally, the study sample was drawn from a national registry of ICU patients with 92% completeness. In the comparison, non-responders were younger than responders at both time points. This could bias our results, as age is significantly associated with fatigue in this study. Another methodological consideration is the use of the CFQ, which is not validated for ICU patients. However, it is considered a generic questionnaire with good psychometric properties and has been used in previous studies involving hospitalised COVID-19 patients (Cella and Chalder, 2010; Bellan et al., 2021; O'Brien et al., 2022). Furthermore, pain is a complex symptom, and we used a simple assessment with the single EQ-5D-5L item regarding pain/discomfort (Devlin et al., 2020). Since this item does not distinguish between pain and discomfort, which can encompass numerous aspects, the results should be interpreted with caution. Future studies should include more comprehensive measures of pain that include multiple dimensions of this complex symptom.

## 5 Conclusions

In this cohort of Norwegian COVID-19 ICU patients, the prevalence of fatigue declined from 3 to 12 months following ICU admission but remained high, with two out of three still reporting fatigue at 12 months. Younger age, pain/discomfort, dyspnoea, and mental health symptoms were all independently and significantly associated with long-term fatigue. The results highlight the complex symptom burden that ICU patients can experience and suggest that clinical follow-up with comprehensive symptom screening is crucial for this patient population.

## CRediT authorship contribution statement

**Kristina Struksnes Fjone:** Writing – review & editing, Writing – original draft, Visualization, Project administration, Methodology, Investigation, Formal analysis, Data curation, Conceptualization. **Milada Hagen:** Writing – review & editing, Visualization, Validation, Supervision, Methodology, Formal analysis, Data curation, Conceptualization. **Jon Henrik Laake:** Writing – review & editing, Visualization, Validation, Methodology, Conceptualization. **Luis Romundstad:** Writing – review & editing, Validation, Methodology, Conceptualization. **Eirik Alnes Buanes:** Writing – review & editing, Writing – original draft, Validation, Supervision, Project administration, Methodology, Funding acquisition, Formal analysis, Data curation, Conceptualization. **Kristin Hofsø:** Writing – review & editing, Writing – original draft, Visualization, Validation, Supervision, Resources, Project administration, Methodology, Investigation, Funding acquisition, Formal analysis, Data curation, Conceptualization.

## Clinical trial registration number

NCT04601090, ClinicalTrials.gov. The trial was registered retrospectively because of the urgent nature of this project.

## Ethics approval statement

This study was performed in line with the principles of the Declaration of Helsinki. The study was approved the Regional Committees for Medical and Health Research Ethics (reference number: 135310) and by the institutional privacy representative.

## Funding

This work was funded by 10.13039/501100004787The Research Council of Norway [grant numbers: 312712/CR, 2020].

## Declaration of competing interest

The authors declare the following financial interests/personal relationships which may be considered as potential competing interests:Kristin Hofso reports financial support was provided by Research Council of Norway. If there are other authors, they declare that they have no known competing financial interests or personal relationships that could have appeared to influence the work reported in this paper.

## Data Availability

The data that support the findings of this study are available from the corresponding author upon reasonable request.
